# Evaluation of Atlantic cod hydrolysate properties in innovative freeze concentration techniques

**DOI:** 10.1016/j.fochx.2025.102325

**Published:** 2025-02-25

**Authors:** Muhammad Umar Khan, Khalid Hamid, Ignat Tolstorebrov, Turid Rustad, Trygve M. Eikevik, Manabu Watanabe

**Affiliations:** a Norwegian University of Science and Technology, Department of Energy and Process Engineering Trondheim, 7491, Norway; b Norwegian University of Science and Technology, Department of Biotechnology and Food Science Trondheim, 7491, Norway; c National University Corporation, Tokyo University of Marine Science and Technology Tokyo, 108, Japan

**Keywords:** Chemical, Thermophysical, Rheological, Differential scanning calorimetry, Fish protein hydrolysates

## Abstract

This study investigates Atlantic cod's (*Gadus morhua*) physicochemical and rheological properties throughout various stages of the hydrolysis process to assess the feasibility of integrating a high-temperature heat pump system for hydrolysis and freeze concentration. This approach will significantly reduce the energy consumption of the current drying processes. The total protein content of cod hydrolysate ranged from 43.38 % ± 0.07 to 87.77 % ± 0.2 on a dry basis, progressing from the initial fish protein hydrolysates mixture to the refined fish protein hydrolysates. The free and total amino acid profiles were analyzed, confirming the presence of all essential amino acids in the samples. Rheology test assessments indicated that fish protein hydrolysates exhibited Newtonian fluid behaviour at lower concentrations, shifting to Bingham fluid behaviour at higher concentrations. Viscosity values spanned from 0.0023 to 1.41 Pa s across concentrations ranging from 3.75 % to 44.42 %. However, deviations in viscosity were observed at elevated temperatures due to decomposition. Differential scanning calorimetry (DSC) determined the FPH's thermal properties and phase transitions concerning water contents. The lowest recorded glass transition temperature was −32.45 °C.

## Highlights

• Investigating the thermophysical and rheological properties of Atlantic cod fish protein hydrolysates.
• Employing differential scanning calorimetry to examine the phase change of fish protein hydrolysates.
• Introducing a novel concentration process for fish protein hydrolysates.
• Highlighting the optimal properties of fish protein hydrolysates for freeze concentration.
• Addressing the limitations associated with freeze concentration.

## Nomenclature

Cod: *Gadus morhua*
d.b: Dry basis
DH: Degree of hydrolysis
DSC: Differential scanning calorimetry
d.w: Dry weight
EC: Evaporation concentration
FPH: Fish protein hydrolysate
FC: Freeze concentration
HPLC: High-performance liquid chromatography
RRMs: Rest raw material
w.b: Wet basis

## 1 Introduction

With projections indicating that the global population will reach 9.7 billion by 2050, there is a rising emphasis on cultivating healthy lifestyles and adopting suitable diets to promote overall well-being (Wang et al., 2021; Willett et al., 2019). This heightened awareness has consequently increased the demand for a sustainable and nutritious food supply (Välimaa et al., 2019). The fish industry is a significant economic source for numerous countries worldwide. Fish is a vital source of protein and plays a crucial role in sustaining populations, particularly in developing nations. Approximately one billion people worldwide rely on fish-related activities, such as production, processing, and trade, for their livelihoods (Oosterveer, 2008).

The fish processing sector generates over 60 % of the total catch as byproducts, encompassing head, skin, trimmings, fins, frames, viscera, and roes. 40 % is directed toward human consumption (Dekkers et al., 2011) while the rest is used for low-value products or even wasted. This substantial byproduct waste seriously threatens environmental well-being and disposal management in developed and developing nations. These by-products contain significant amounts of protein-rich material and lipids that could be repurposed as valuable market products, including food, animal feed, fish meal, and fertilizer (Hsu, 2010).

Various bioprocesses have been developed to extract essential nutrients and bioactive compounds to harness the protein-rich by-products of fish processing. These nutrients enhance human health by safeguarding against diseases, supplying vital nutrients, and, at the same time, addressing pollution and disposal challenges. Among the current biotechnological processes employed, enzymatic hydrolysis of fish proteins is among the most popular and effective. This process yields biologically active protein hydrolysates from commercial fish by-products and underutilized fish rest raw materials, unlocking their nutritional and physiological benefits (Bartolomei et al., 2023; Hsu, 2010; Je et al., 2009; Ngo et al., 2010; Raghavan & Kristinsson, 2008; Ren et al., 2008; Samaranayaka & Li-Chan, 2008; Wijayanti et al., 2016).

Fish protein hydrolysates (FPH) are produced from fish or fish by-products through protein hydrolysis, breaking the proteins in fish tissues into smaller components, including peptides and amino acids. As a result, FPH is a mixture of fragmented proteins, offering a concentrated source of these smaller protein units(Damodaran et al., 2007; Petrova et al., 2018). Due to the breakdown of larger proteins into smaller peptides, improved functional properties were reported by He et al. (2013) and Kristinsson and Rasco (2000) when comparing the FPH to the original protein. The techno-functional characteristics of FPH, encompassing attributes like water and oil binding capacity, solubility, freezing and melting temperatures, and viscosity, depend on the raw material type and various process parameters.

The parameters influencing the process include the type of raw material, the proteases employed, pH levels, reaction temperature, and the duration of hydrolysis (Baharuddin et al., 2015; Leni et al., 2020). Additionally, the functional properties of FPH are influenced by characteristics, including the degree of hydrolysis (DH), the size, shape, amino acid sequence, and composition of peptides, and the presence of ions in the mixture. The DH is a critical monitoring metric for the process of hydrolysation. It can be employed as an indicator to compare different protein hydrolysates (Baharuddin et al., 2015; Leni et al., 2020). Other properties, like the anti-oxidative and anti-hypertensive behaviour of FPH, have been shown to be enhanced, as documented in various studies (Chalamaiah et al., 2012; He et al., 2013). Jenkelunas and Li-Chan (2018) studied and demonstrated that FPH can be used in cryoprotectant applications for aquatic food products that are in a frozen state.

Liquid FPH is a water mixture of hydrolysed proteins, and the original product contains 84 to 95 % water content (G. A. Gbogouri et al., 2004a; Nilsang et al., 2005; Ovissipour et al., 2009).Liquid FPH is highly unstable. Additionally, transportation costs are high because of strict cold chain requirements. Consequently, dried FPH is preferred because it offers a longer shelf life, more affordable storage, and lower transportation costs. At the same time, drying FPH is a complicated and energy-demanding process (Petrova et al., 2018). Conventionally, the spray dryer removes the moisture content and converts FPH to a dried powder state. The industrial spray dryer shows energy consumption between 4500 and 11,500 kJ/kg of water removed (Mujumdar, 2007).To minimize energy consumption in the spray drying process, FPH is concentrated through evaporation or reverse osmosis (RO) before being introduced into the spray dryer.

Evaporation occurs under vacuum within a temperature range of 50 °C to 100 °C. However, food products are typically sensitive to heat, and the elevated temperatures in evaporation can lead to various issues. Most significantly, nutrients, flavors, and colors may be compromised, resulting in reduced overall product quality (Berk, 2016; Giner Santonja, 2020; Polydera et al., 2003). RO is a non-thermal concentration process widely used in the food industry. It applies pressure to force water through a semipermeable membrane, separating it from dissolved solids and other impurities. However, a single RO system typically achieves a maximum concentration level of only 25–30 % *w*/w. The drawbacks of RO plants are significant, including fouling issues, loss of aroma, challenges in concentrating solutions with highly suspended solids, and the high costs associated with operation (Berk, 2016; Jesus et al., 2007; Jiao et al., 2004).

Freeze concentration (FC) represents a nonthermal method of concentration that addresses the limitations of evaporation and RO. In this process, specific amounts of water in aqueous solutions are frozen, forming pure ice crystals. Subsequently, these ice crystals are separated from the liquid phase, allowing the remaining solution to be concentrated effectively. The FC process is usually applied to the entire initial volume of liquid. This approach ensures maximum separation of water as ice crystals, leading to a higher recovery of valuable components in the hydrolysis solution. Although processing a smaller volume would concentrate the solution, it would reduce the overall yield, making the process less efficient and less practical for industrial-scale applications. This strategy highlights the efficiency and scalability of the FC process in achieving high product recovery while maintaining energy efficiency.

FC provides significant advantages in producing high-quality concentrates compared to evaporation concentration (EC) and membrane technology. The most apparent benefit of FC lies in its ability to operate at low temperatures without a vapor/liquid interface. Moreover, the use of FC preserves nutrients, flavors, and colors in the concentrated product, ensuring the retention of the original qualities of the solution (Benedetti et al., 2015; Khan et al., 2024; Miyawaki et al., 2016; Moreno et al., 2014; Morison & Hartel, 2006).Furthermore, it's noteworthy that the latent heat of water freezing (334 kJ/kg) is approximately one-seventh of the enthalpy required for water vaporization (2260 kJ/kg). This substantial difference in energy requirements highlights the significant energy-saving potential of the FC process (Miyawaki et al., 2005; Pazmiño et al., 2017). Numerous studies have explored FC applications for a range of fruit juices, such as kiwi juice (Maltini & Mastrocola, 1999), berry juices (Di Cesare et al., 2000; Ghizzoni et al., 1995), and apple and pear juices (Hernández et al., 2010; Nazir & Farid, 2008), with citrus fruit juices (Braddock & Marcy, 1987; Van Nistelrooij, 2005) being the most common application. Beyond fruit juices, FC is utilized in various other food industries, including dairy products (Hartel & Espinel, 1993; Van Nistelrooij, 2005), brewing (Putman et al., 1997), winery and distilling (Di Cesare et al., 1993), and concentrating dilute solutions of tea and coffee.

This study comprehensively analyzed the physicochemical and rheological properties of FPH and FPH mixture derived from cod (*Gadus morhua*) across different production and concentration processes. Detailed insights into these thermal and rheological characteristics are essential for effectively designing and optimizing high-temperature heat pumps and various concentration methods and equipment, including FC, evaporation, and reverse osmosis.

## 2 Material and methods

### 2.1 Preparation of samples

**Fig. 1:**
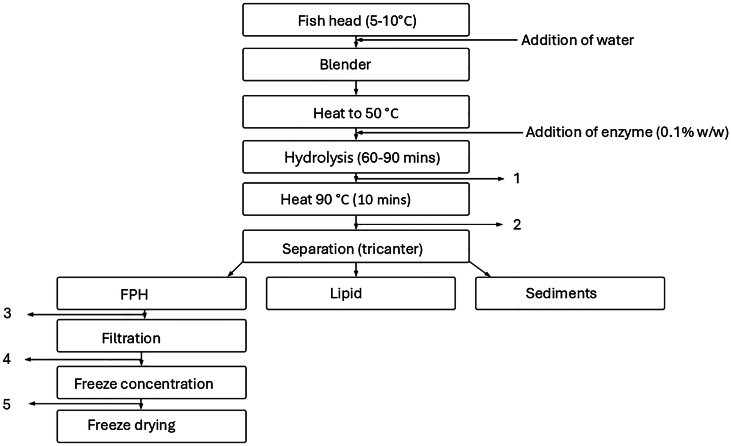
The schematic diagram for the hydrolysis and drying process.

Using enzymatic hydrolysis, our partner company produced cod hydrolysates from cod heads. The enzyme used was a mixture of papain and bromelain (1:1), 0.1 % of the raw material weight. Samples were collected at various stages throughout hydrolysis, as depicted in Fig. 1, indicating the specific collection points (Table 1). Samples were collected at every depicted stage and subjected to freeze-drying as described in **Section 2.2**. These samples were systematically analyzed at each process stage to evaluate their thermophysical and rheological properties and assess the feasibility of implementing a high-temperature heat pump.

**Table 1:** Raw material type and sample names.

**Type of raw material**	**Temperature (°C)**		**Post-processing**	**Sample names**
Cod heads	50	1	–	FPH mixture 50 °C
	90	2	–	FPH mixture 90 °C
	–	3	Before filtration	FPH before filtration
	–	4	After filtration	FPH after polishing
	–	5	FC	FC (17 ± 0.2 %) FC (24 ± 0.1 %)

Cod hydrolysates were separated using a tricanter with a 1 ton/h capacity, followed by FC to achieve various concentration levels. The FC process was conducted on FPH after polishing at two refrigeration temperatures: −10 °C and − 15 °C. At −10 °C, a concentration level of 17 % was achieved with 67.2 % ice formation, while at −15 °C, a concentration level of 24 % was achieved with 76.2 % ice formation. Each FC run lasted one hour, allowing for efficient separation of water as ice while preserving the quality of the hydrolysates. These variables were optimized to balance energy efficiency, concentration yield, and sample integrity.

The concentrated samples were subsequently freeze-dried (**Section 2.2**) to generate samples for analysis. Although freeze-drying is not typically employed for industrial food- or feed-grade fish protein hydrolysates, it was utilized here to ensure precise experimental control and produce high-quality samples for detailed characterization. To ensure accuracy, each sample was tested in triplicate to minimize error.

### 2.2 Freeze drying of fish protein hydrolysates

FPH was produced in a liquid state, and the liquid samples were placed in small plastic bags and slowly frozen at −20 °C for two days until solidified. Subsequently, the samples were transported to a laboratory freeze dryer, where a drying pressure of 0.3 mbar was applied, accompanied by a 200-watt heat supply for 22 h. After drying, the resulting dried samples were pulverized into a powder form and vacuum-sealed in low-density polyethylene bags. The polyethylene bags were stored on dark shelves at 22 °C.

### 2.3 Degree of hydrolysis

The extent of the DH was assessed through formol titration, quantifying the percentage of unbound amino groups relative to the sample's overall nitrogen content, as outlined in Taylor (1957):

(1) $$\frac{A*B*14.007*100}{C*100}=\text{free amino groups}\;\left(\%\right)$$

A = ml NaOH used.

B = Concentration of the NaOH solution used in titration (0.1 M).

C = weight of the sample (g).

The DH was subsequently determined by dividing the quantity of free amino groups by the total nitrogen content, which was calculated based on the concentration of total protein (percentage of protein divided by a factor of 6.25).

### 2.4 Protein content

The protein content of the freeze-dried samples was determined using the C/N/S elemental analysis method. A C/N elemental analyzer (Elementar Combustion System CHNS-O, ECS 4010, Langenselbold, Germany) was used to determine the total nitrogen content in the samples. A protein conversion factor of 6.25 was used.

### 2.5 Protein solubility

Protein extracts were prepared by dissolving 0.5 g of each FPH sample in 10 mL of distilled water. The solutions were homogenized and centrifuged. Water-soluble proteins were determined in triplicates by using the Lowry et al. (1951). Bovine serum albumin (BSA) was used to prepare a standard curve.

### 2.6 Total amino acid composition

The analysis of total amino acid content was done as described by Blackburn (1978).Samples were hydrolyzed in 6 M hydrochloric acid (HCl) for 22 h at 105 °C in airtight vials to limit oxidation, neutralized with sodium hydroxide, and filtered through Whatman glass microfiber filter GF/C by vacuum. The samples were diluted with de-ionized water and filtered through a 0.22 μm syringe filter. Analysis was done by reverse phase HPLC (UltiMate 3000 HPLC, Thermo Scientific, USA) using a Waters Nova-Pak C18 column with methanol and sodium acetate as eluents. The detector was a Dionex RF 2000 fluorescence detector with OPA derivatization. The column temperature was 25 °C. GLY/ARG were determined together as their peaks merged.

### 2.7 Amount and composition of free amino acids

The determination of free amino acids in the freeze-dried raw material and hydrolysates was performed according to Osnes and Mohr (1985). Two parallels of each sample were made by transferring 1 ml of water-soluble protein extract (0.5 g sample in 10 ml doubly distilled water) into EPPENDORF tubes and added 0.25 ml of 10 % sulphosalisylic acid. The samples were shaken to mix the solutions evenly. Next, they were placed into a cold room at 4 °C. After 30 min, the samples were centrifuged with an EPPENDORF 5415 R centrifuge (Eppendorf AG, Germany) for 10 min at 10,000 g. The supernatant was diluted and filtered. 0.25 ml of each filtered sample was pipetted into vials and analyzed by HPLC in the same way as total amino acids.

### 2.8 Ash

The ash content was determined by combusting the dried samples in a muffle furnace at 550 °C for 12 h (Standardization, 2002).

### 2.9 Solid content

Each sample was placed on a pre-weighed aluminum plate, and the combined weight of the plate and sample was recorded. The plate was then placed in an oven at 100 °C and left overnight. After 24 h, the plate was removed, and the weight of the dried sample was measured. The solid content was determined by calculating the difference in sample weight before and after drying.

### 2.10 Molecular weight distribution

Gel filtration was performed using a fast protein liquid chromatography (FPLC) system to analyse the molecular weight distribution of the hydrolysates. A sample of 0.1 g was dissolved in a 0.05 M acetate buffer (4 mL) with a pH of 5 and separated on a SuperdexTM Peptide 10/300 GL column. This column separates peptides within a molecular weight range from 67,000 to 1000 Da.

For calibration and reference, the following standards were employed:

• Cytochrome c (Molecular Weight: 12,327)
• Vitamin B12 (Molecular Weight: 1355)
• BSA (Molecular Weight: 66,430)

The samples were run at room temperature on an Äkta start (cytiva.com) system using a Superdex 75PG column (16 mm diam, height 12 cm), with a 0,2 M NaAcetate buffer, rate 0,5 ml/min, detection UV 280 nm. Samples of 100 ul with a concentration of 1 mg/ml were applied to the column.

### 2.11 Viscosity of fish protein hydrolysates

The study utilized a Peltier plate with a diameter of 40 mm, and precise measurements required a sample volume of 0.13 ml, with 1000 μm between the peltier plate and the bottom surface. The shear rate was systematically varied from 10 s^−1^ to 100 s^−1^, and the corresponding shear stress was subsequently measured.

In addition to rheometers, viscosity cups (Industrial physics, USA) were employed in this study. Flow through an orifice is a commonly used technique for assessing and categorizing viscosity. This method allows for determining kinematic viscosity, represented by the flow time measured in seconds. A viscosity disc calculator or table can convert the resulting values into centistokes. Viscosity flow cups are valuable tools for evaluating the consistency of FPH at various concentrations and temperatures. By employing these methods, the study aimed to comprehensively analyse the viscosity of FPH and gain insights into their flow behaviour under different conditions.

The Herschel Bulkley model finds application in describing viscosity changes concerning the concentration of dry matter content Table 5. The following section outlines the models:

(2) $$\tau =\tau_{0}+k{\left(\gamma \right)}^{n}\;$$

Where τ is shear stress, Pa; τ_0_ – yield stress, Pa; k – consistency index, Pa*s^n^; γ- shear rate, s^−1^; n*- flow index.

To prepare samples for viscosity measurements, the freeze-concentrated hydrolysates (FC 17 %, FC 24 %) and the polished FPH were first dried using a vacuum dryer as outlined in section **2.2**. The dried samples were then reconstituted by adding water to achieve the desired dry matter concentrations, as specified in Table 5 for viscosity analysis. This method ensured precise control over the sample composition and allowed for consistent rheological evaluation.

### 2.12 Differential scanning calorimetry

The differential scanning calorimetry (DSC) analysis was conducted using a DSC Q2000 instrument (TA Instruments, USA) equipped with a liquid nitrogen cooling system also provided by TA Instruments. Indium was used for temperature and cell calibration. The heating capacity was calibrated using a sapphire within the temperature range of −150 °C to 150 °C. Following TA's instrument guideline, helium was selected as the purge gas at a 25 mL/min flow rate. For the reference sample, an aluminum pan was used and hermetically sealed.

Samples containing varying moisture content were prepared by dissolving the dehydrated FPH powder in distilled water. The water content of each sample was assessed using the following procedure: After obtaining DSC results, all hermetically sealed pans were punctured with a needle and subsequently dried at 105 °C until a constant weight was achieved. The water content was then calculated as the ratio of the weight difference between the pans before and after the drying process.

#### 2.12.1 Determination of the glass transition, freezing, and melting temperatures

The DSC heating curve analysis determined the onset of ice melting (T_m_) and the conclusion of freezing. The freezing point was defined as the minimum value of the ice melting endothermic peak on the DSC heat flow curve in its second derivative concerning temperature. The glass transition temperature was ascertained using TA Universal Analysis 2000 version 4.5 A software (TA Instruments, USA). The onset and conclusion of the glass transition were identified by extrapolating the baselines to the point of intersection with the glass transition line. Additionally, the glass transition's (T_i_) inflection point was determined by deriving the heat flow curve concerning temperature, following the method described by Tolstorebrov et al. (2014b).

#### 2.12.2 Determination of unfreezable water

Unfreezable water content was determined by analysing the DSC melting curve. The DSC melting peaks were integrated using the sigmoidal tangent baseline function. To estimate the mass of ice (I, in kilograms) through the conventional method, the melting energy of ice in the fish (E_fish_) was divided by the heat of fusion of pure ice (L_ice_) (Kumagai et al., 1985):

(3) $$I=\frac{E_{\text{fish}}}{L_{\mathrm{ice}}}$$

L_ice_ = 333.5 kJ/kg at 0 °C. The enthalpy of ice melting is contingent upon the temperature of melting, T_m_, and freezing point (T_f_). The empirical formula suggested by Riedel (1978) was used:

(4) $$L_{\mathrm{ice}}=333.5+2.05*T_{m}-4.19*10^{-3}{T^{2}}_{m}$$

Eq. (5) estimated the ice fraction (Eq. (3) modified by Eq. (4)- modified method:

(5) $$x_{\mathrm{ice}}=\frac{\int_{T}^{T_{f}}\mathrm{IdT}}{\int_{T_{m}}^{T_{f}}\mathrm{IdT}}$$

With dT of 0.02 °C, determining unfreezable water involved calculating the discrepancy between the product's total water fraction and its ice fraction.

## 3 Result and discussion

### 3.1 Chemical composition

The chemical composition of FPH is approximately determined by factors such as the composition of raw materials, the specificity of the enzyme used, and the conditions under which hydrolysis occurs. The proximate composition of different samples collected at each step of the hydrolysis process in Fig. 1 is presented in Table 2. The first two samples, collected after hydrolysis at 50 °C and after heating to 90 °C after hydrolysis, contain protein, ash, and sediments. The tricanter effectively separates the ash and sediments, resulting in the third sample, which includes both soluble and insoluble protein content. Following filtration, the subsequent samples contain primarily water, the soluble fraction of protein, and trace amounts of ash and minerals.

**Table 2:** Chemical composition, degree of hydrolysis, and solid content.

**Sample name**	**Protein (d.b%)**		**Ash (d.b%)**	**Degree of hydrolysis**	**Solid content (w.b%)**
	Soluble	Total			
FPH mixture at 50 °C	32.33 ± 0.2	49.08 ± 5.4	3.27 ± 0.013	19.06 ± 0.46	25.61 ± 0.83
FPH mixture at 90 °C	31.12 ± 0.4	43.38 ± 0.07	3.38 ± 0.07	14.25 ± 0.12	25.61 ± 1.2
FPH before filtration	43.18 ± 0.4	76.65 ± 0.02	9.80 ± 0.18	21.69 ± 0.07	10.61 ± 0.53
FPH after polishing	87.17 ± 0.1	87.77 ± 0.2	7.89 ± 0.0007	18.45 ± 0.17	5.8 ± 0.5
FC (17 ± 0.2 %)	74.74 ± 0.3	75.13 ± 0.5	13.13 ± 0.00092	20.15 ± 0.16	17 ± 0.2
FC (24 ± 0.1 %)	74.89 ± 3.44	76.52 ± 0.45	13.2 ± 0.04	20.48 ± 0.284	24 ± 0.1

According to Remme et al. (2022), the high ash concentration in FPH may be attributed to the abundant bone substance in cod heads. The ash content in FPH ranges from 3.27 % to 13.24 % d.b, and it exhibits variations throughout the hydrolysis process. Notably, the lowest ash content is observed in the FPH mixture at 50 °C and 90 °C, while the concentrated sample (24 ± 0.1) after FC contains the highest ash content. The reduction in ash concentration during hydrolysis can be attributed to separating two phases: salts and other minerals dissolve into the water phase, while bone fragments settle as sediments. These sediments are subsequently removed through the separation and filtration process (Remme et al., 2022).

Implementing a heat pump requires the precise design of heat exchangers, necessitating a thorough understanding of critical parameters such as heat capacity, thermal conductivity, and solution density. The proximate chemical composition of the solution directly influences these thermophysical properties. As shown in Table 2, the solid content of samples at various process stages reveals that the highest solid content is achieved when the FPH mixture is heated to 90 °C. The highest solid content in the FPH mixture observed at 90 °C can be explained by its initial composition, which includes bones, ash, and both soluble and insoluble protein content. During hydrolysis, these components are separated, leaving only the soluble portion, which decreases the solid content. The other ingredients are separately processed, accounting for the reduction in solid content in subsequent steps.

Moreover, heating the mixture to 90 °C leads to partial evaporation of water, which concentrates the remaining solids. This combined effect of initial composition and water loss during heating contributes to the observed solid content. Notably, water is the predominant component in all samples, significantly impacting their thermophysical properties, making them ideal considerations for heat exchanger design.

Samples were vacuum-dried before chemical composition analysis, and the protein content in FPH was categorized into soluble and total fractions, with total protein content ranging from 43.38 % to 87.77 % d.b. The lowest protein content was observed in the FPH mixture heated to 90 °C, while the highest protein content was found in the polished FPH. These results are consistent with those reported by Remme et al. (2022) and G. Gbogouri et al. (2004). Interestingly, the FPH mixture after hydrolysis contained the lowest soluble protein content, whereas the polished FPH had the highest.

### 3.2 Degree of hydrolysis

The size of the peptide molecule significantly influences the surface activity and bioactivities of FPHs, and the DH also plays a pivotal role in determining the surface activity and bioactivities of FPHs (Jeon et al., 2000; Kristinsson & Rasco, 2000). Additionally, the DH is a significant factor as it refers to the proportion of cleaved peptide bonds within a protein hydrolysate. Solubility is another essential consideration for the utility of FPHs as a food ingredient. Studies have shown that the solubility of FPHs increases as DH increases (G. A. Gbogouri et al., 2004b; Jamdar et al., 2010). However, it is essential to note that a higher DH can result in bitter peptides, negatively impacting palatability. Moreover, excessively high DH levels can adversely affect the functional properties of FPHs (Kristinsson & Rasco, 2000).

Table 2 displays the fluctuations in DH across all the samples. DH varied from 14.25 % to 21.69 %. FPH mixture after heating at 90 °C has the lowest degree of hydrolysis, while the FPH has the highest DH before filtration. Reports indicate that an increased DH enhances protein recovery since the cleavage of more peptide bonds leads to protein hydrolysates with lower molecular weights, which are more soluble in water (He et al., 2013).

The DH reflects the extent of enzymatic hydrolysis and is expected to remain consistent across samples collected after the same hydrolysis stage. However, the observed variation in the DH of the FPH mixture at 90 °C may be attributed to the cessation of enzymatic activity due to heat inactivation at elevated temperatures. At 90 °C, protein denaturation could lead to the formation of aggregates or complexes, potentially interfering with the measurement of DH. These aggregates may alter the solubility or reactivity of the hydrolyzed peptides, resulting in slight variations in the measured DH values.

### 3.3 Amino acid

Table 3 and Table 4 show the content of free and total amino acids in different stages of the hydrolysis and concentration process. Free amino acids, including Gln, Lys, Arg, Ala, Leu, Asp, and Asn, were produced by the action of enzymes used during hydrolysis.

**Table 3:** Free amino acid profile (mg/g d.w).

	**FPH mixture after 50** **°C**	**FPH mixture after 90** **°C**	**FPH before filtration**	**FPH after filtration**	**FC (17 ± 0.2 %)**	**FC (24 ± 0.1)**
Asp	1.06 ± 0.01	0.6 ± 0.02	6.10 ± 0.001	1.68 ± 0.05	3.69 ± 0.05	4.203 ± 0.134
Glu	3.45 ± 0.03	1.6 ± 0.04	15.6 ± 0.07	4.64 ± 0.05	5.34 ± 0.009	6.65 ± 0.65
Asn	0.07 ± 0.0007	0.02 ± 0.001	0.18 ± 0.002	0.09 ± 0.0006	0.01 ± 0.001	0.01 ± 0.00
His	1.53 ± 0.05	0.74 ± 0.026	0.78 ± 0.02	2.17 ± 0.02	1.12 ± 0.08	1.34 ± 0.19
Ser	2.46 ± 0.05	0.91 ± 0.0133	5.46 ± 0.02	2.86 ± 0.094	0.81 ± 0.07	5.01 ± 0.19
Gln	3.80 ± 0.01	1.56 ± 0.077	3.37 ± 0.01	3.78 ± 0.043	2.69 ± 0.013	2.75 ± 0.171
Gly/Arg	5.48 ± 0.006	1.79 ± 0.12	7.17 ± 0.3	4.93 ± 0.11	11.06 ± 0.2	11.83 ± 0.134
Thr	2.72 ± 0.023	0.92 ± 0.074	8.25 ± 0.14	2.53 ± 0.094	3.35 ± 0.05	3.29 ± 0.71
ALa	11.44 ± 0.01	4.90 ± 0.0091	16.22 ± 0.023	13.37 ± 0.03	16.18 ± 0.01	17.65 ± 0.47
Tyr	0.50 ± 0.015	0.45 ± 0.13	1.82 ± 0.11	1.21 ± 0.03	1.51 ± 0.13	1.87 ± 0.17
Aba	0.02 ± 0.004	0.05 ± 0.0036	1.02 ± 0.012	0.14 ± 0.004	0.49 ± 0.01	0.59 ± 0.0361
Met	3.68 ± 0.04	1.19 ± 0.005	4.39 ± 0.01	3.64 ± 0.02	4.52 ± 0.05	5.15 ± 0.145
Val	2.92 ± 0.013	0.94 ± 0.026	4.97 ± 0.02	2.69 ± 0.031	3.47 ± 0.008	3.94 ± 0.038
Phe	3.22 ± 0.021	0.17 ± 0.024	4.08 ± 0.01	3.76 ± 0.06	3.95 ± 0.023	4.44 ± 0.0862
Ile	2.35 ± 0.005	0.64 ± 0.066	3.18 ± 0.06	1.74 ± 0.094	2.69 ± 0.07	2.94 ± 0.0624
Leu	5.57 ± 0.042	2.67 ± 0.023	10.26 ± 0.05	8.03 ± 0.132	7.09 ± 0.06	9.36 ± 1.8
Lys	2.52 ± 0.011	1.38 ± 0.073	5.44 ± 0.084	3.78 ± 0.14	5.15 ± 0.03	5.74 ± 0.15
Total	52.80 ± 0.06	21.50 ± 0.489	98.28 ± 0.8	61.06 ± 0.94	77.12 ± 0.5	86.71 ± 3.04

**Table 4:** Total amino acid (mg/g d.w).

	**FPH mixture after 50** **°C**	**FPH mixture after 90** **°C**	**FPH before filtration**	**FPH after polishing**	**FC (17 ± 0.2)**	**FC (24 ± 0.1)**
Asp	26.36 ± 4.72	32.37 ± 2.43	40.95 ± 0.80	65.99 ± 5.51	34.80 ± 3.60	45.88 ± 5.88
Glu	34.70 ± 2.70	59.50 ± 19.50	66.52 ± 0.0011	185.75 ± 11.60	46.20 ± 3.30	64.27 ± 8.71
Asn	0.00 ± 0.00	0.02 ± 0.01	0.02 ± 0.003	0.4 ± 0.02	0.02 ± 0.01	0.02 ± 0.01
His	5.27 ± 0.80	7.22 ± 0.10	9.22 ± 0.09	10.06 ± 1.12	4.21 ± 0.50	8.22 ± 1.42
Ser	11.40 ± 2.40	14.70 ± 0.34	25.18 ± 0.43	26.57 ± 2.42	20.36 ± 2.30	29.59 ± 4.98
Gln	2.01 ± 0.25	3.59 ± 0.19	0.05 ± 0.05	6.16 ± 0.41	0.29 ± 0.03	0.677 ± 0.28
Gly/Arg	61.39 ± 5.63	67.93 ± 0.22	72.17 ± 1.10	152.00 ± 11.50	95.35 ± 6.70	126.53 ± 19.65
Thr	13.14 ± 1.03	17.17 ± 0.07	28.13 ± 0.12	27.07 ± 2.11	15.38 ± 0.73	21.86 ± 4.02
Tyr	20.50 ± 2.01	22.15 ± 0.13	32.53 ± 4.93	48.80 ± 6.72	28.18 ± 1.77	36.14 ± 4.76
Ala	4.34 ± 3.86	8.95 ± 0.55	7.67 ± 0.66	4.91 ± 4.20	1.58 ± 3.13	8.56 ± 1.33
Aba	0.20 ± 0.07	0.20 ± 0.10	3.54 ± 0.58	0.15 ± 0.08	0.38 ± 0.06	0.453 ± 0.038
Met	7.66 ± 1.54	7.38 ± 0.15	10.64 ± 1.50	16.74 ± 2.55	10.61 ± 1.03	14 ± 1.751
Val	11.26 ± 2.01	11.25 ± 0.03	22.81 ± 0.97	19.42 ± 1.94	13.46 ± 1.36	16.9 ± 2.166
Phe	10.72 ± 1.83	11.42 ± 0.40	1.51 ± 0.36	19.01 ± 1.83	12.83 ± 1.31	16.43 ± 2.176
Ile	9.52 ± 1.70	8.56 ± 0.06	15.14 ± 0.80	14.96 ± 1.40	10.80 ± 1.10	13.387 ± 1.375
Leu	20.24 ± 3.62	20.56 ± 0.21	39.19 ± 1.51	36.64 ± 5.56	25.82 ± 2.73	33.24 ± 4.51
Lys	21.14 ± 3.97	24.19 ± 0.21	49.86 ± 1.58	46.32 ± 3.96	26.56 ± 2.87	34.89 ± 5.2
Total	259.87 ± 47.45	306.10 ± 34.22	423.78 ± 9.94	666.54 ± 84.90	336.21 ± 66.42	462.32 ± 61.61

Research indicates that many of these amino acids possess cryoprotective effects in actomyosin systems (Jiang et al., 1987). Nonetheless, it should be highlighted that the content of free amino acids varies during the hydrolysis and concentration process; the highest free amino acid is exhibited in FPH before filtration.

It has been proposed that small peptides may work as effective antifreeze agents to diminish the ice crystal growth rate, thereby addressing potential obstacles in the FC process (Kim et al., 2009). Cheung et al. (2009) documented that peptides, instead of free amino acids, could significantly contribute to the cryoprotective activity observed in FPH. However, this needs to be further studied. Future work will aim to investigate the peptide profiles in greater detail, employing advanced analytical techniques such as mass spectrometry to confirm the presence of tetrapeptides and their potential contribution to cryoprotective properties.

The amino acid composition of cod samples changes during the hydrolysis process. When the FPH mixture is heated up to 90 °C, the free amino acid content reaches a minimum value of 21.50 ± 0.489 mg/g. Similarly, the total amino acid profile reaches its minimum value when the FPH mixture is heated to 50 °C, measuring 260 ± 47 mg/g. On the other hand, FPH before filtration exhibits the highest free amino acid content at 98.3 ± 0.8 mg/g, while FPH, after polishing, displays the highest total amino acid content at 667 ± 85 mg/g.

### 3.4 Molecular weight distribution

Due to enzymes targeting specific cleavage points on polypeptide chains, the resultant FPH products comprise peptide molecules of varying lengths (Barkia et al., 2010). This research employed gel filtration to separate FPH and analyse their peptide size distribution. The chain length of peptides, influenced by DH, holds particular importance concerning traits like bitterness, emulsion capacity, and solubility (G. A. Gbogouri et al., 2004).

Prior research indicates an optimal molecular size or chain length for peptides that guarantees effective foaming and emulsifying characteristics. Additionally, it suggests that extensive hydrolysis leading to smaller peptide molecules diminishes these properties (Jeon et al., 2000; Kristinsson & Rasco, 2000; Šližytė et al., 2009). FPH mixture at 90 °C exhibited the highest molecular weight compared to other samples. This is because this sample contains both soluble and insoluble fractions. In the separated samples, the main part of the peptides has a molecular weight of around 1000 Da.

### 3.5 Viscosity

The viscosities of FPH were measured using two approaches: electrometers and cups. The electrometer-based measurements determined viscosity by evaluating the ratio between shear stress and shear rate, while the cup-based measurements were flow-rate-based.

However, the viscosities of samples before filtration and hydrolysis, such as the FPH mixture at 50 °C and 90 °C, could not be accurately measured due to the presence of suspended particles. When heated using Peltier plates, these samples developed a crust on the surface, which disrupted the shear stress measurements. Due to these limitations, these samples were omitted from Table 5. Similarly, the remaining samples displayed the same behaviour when heated above 50 °C. This was due to protein denaturation, which caused the formation of a surface crust, disrupting viscosity measurements and leading to inconsistencies in the data.

**Table 5:** Rheological properties of FPH concerning dry matter content.

**Sample name**	**Concentration (%)**	**Shear rate (s**^**−1**^**)**	**6** **°C**		**25** **°C**		**50** **°C**	
			**k**	**τ**_**0**_	**k**	**τ**_**0**_	**k**	**τ**_**0**_
FC (17 ± 0.2 %), FC (24.1 ± 0.1 %) and FPH after polishing.	3.75	50–100	0.0023 ± 0	0	0.0016 ± 0.00006	0	0.0011 ± 0	0
	9.12		0.0039 ± 0.00006	0	0.0028 ± 0.00006	0	0.0018 ± 0.00006	0
	20		0.016 ± 0.00047	0	0.0117 ± 0.00017	0	0.0067 ± 0.000153	0
	27.03		0.0278 ± 0.000473	0	0.022 ± 0.00023	0	0.0115 ± 0.000058	0
	33.30		0.068 ± 0.00085	0	0.0553 ± 0.0011	0	0.0286 ± 0.000265	0
	35.97		0.096 ± 0.0021	0	0.0835 ± 0.000153	0	0.0408 ± 0.000361	0
	44.42		1.15 ± 0.06	18.77 ± 6.79	0.48 ± 0.113	17.1 ± 4.4	0.43 ± 0.188	5.66 ± 1.91

*n = 1.

**Fig. 2:**
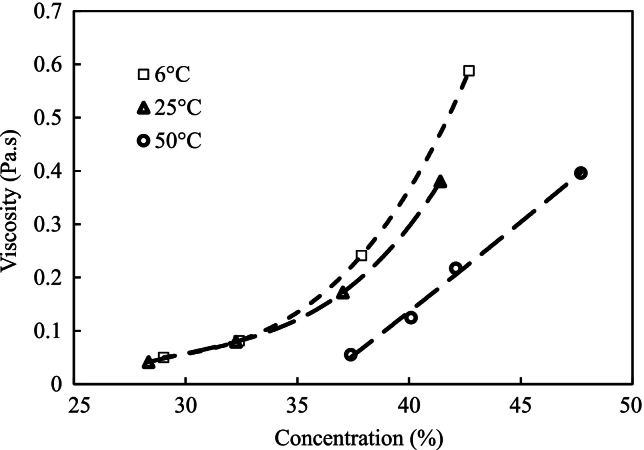
Viscosity measured from viscosity cups from cod hydrolysates (FC (24 ± 0.1 %)).

FPH with dry matter below 44 % w.b. demonstrates typical Newtonian behaviour, characterized by a constant viscosity regardless of fluid flow velocity. This behaviour is comparable to water, oils, and other fluids. However, as the dry matter content increases, FPH exhibits non-Newtonian behaviour, showing yield stress and adopting typical Bingham plastic liquid characteristics (Fig. 2). Research on FPH from stone fish recently demonstrated that viscosity did not vary with flow velocity at low concentrations. At the same time, it varied between 0.003 and 0.04 Pa s for concentrations ranging from 1.0 % to 10 % *w*/*v*, respectively (Auwal et al., 2017).

FPH displayed another intriguing behaviour during flow cup experiments. At high temperatures, the FPH tends to adhere to the orifice, causing a hindrance in the flow. This phenomenon can also be observed in pipe systems, highlighting the importance of using larger pipes for transportation at elevated temperatures to mitigate flow obstruction. Both studies also identified the presence of shear thinning behaviour at high concentrations, which aligns with findings from previous studies on non-FPH, such as soy protein hydrolysate (Lamsal et al., 2007) and milk protein hydrolysate (Gruppi et al., 2022). This indicates a consistent trend across different types of protein hydrolysates.

An empirical model has been formulated to predict the viscosity of FPH in relation to concentration levels. Developed using a regression approach, the model is as follows:

(6) $$\text{Viscosity}=9.73*10^{-6}*\mathrm{tem}p^{-0.48}*1.33^{\text{conc}}$$

**Fig. 3:**
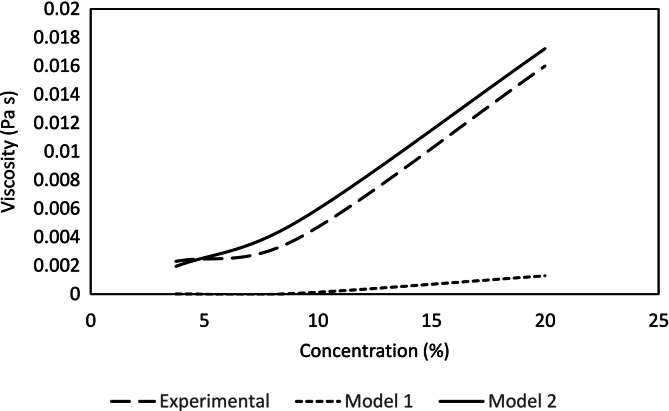
Comparison of different models.

Where temp is the temperature in °C, and conc is the concentration of solid contents in percentage. Fig. 3 illustrates the comparison of experimental data with the viscosity model at 6 °C. The model 1 shows a significant deviation from experimental data for concentrations below 20 %, indicating poor predictive performance in this range.

A new model, developed for concentration levels below 20 %, is provided below:

(7) $$\text{Viscosity}=1.35*10^{-3}+8.55*10^{-6}*\text{temp}+8.1*10^{-5}*\text{conc}+4.63*10^{-7}*{\text{temp}}^{2}+3.55*10^{-5}*{\text{conc}}^{2}-1.2*10^{-5}*\text{temp}*\text{conc}$$

The figure below shows both the models and how the second models predict the viscosity of FPH more accurately compared to 1st model.

The FC method encounters significant limitations that define the highest attainable concentration. These constraints primarily hinge on the viscosity of the mother liquor and the extent of freezing point depression (Ruemekorf, 2000). As the freezing point decreases, a more sophisticated refrigeration system is essential to generate the necessary cooling. Conversely, with an increase in viscosity, various challenges emerge. Initially, it becomes difficult to pump a viscous solution through the equipment. Furthermore, as viscosity reaches a specific threshold, it impedes the growth of ice crystals, indicating the maximum concentration point (van Beek et al., 2018).

Increased viscosity decelerates crystal growth, requiring prolonged residence times and larger-scale equipment to achieve distinguishable crystal sizes. This necessity arises from the interrelation between ice separator capacity and viscosity, where higher viscosity inversely affects the capacity (Braslavsky, 2015; Petzold & Aguilera, 2009).

Adhering to prevalent industrial standards, the bulk temperature of the mother liquor is typically restricted to −5 °C, while the viscosity usually falls within the range of 150–400 mPa s, as documented by van Beek et al. (2018) and Severine Dette (2020). Based on our research findings and extrapolating viscosity and freezing point data, it is projected that the maximum concentration achievable by FC would be approximately 43 %. At this concentration, the viscosity of FPH is approximately 300 mPa s, and the freezing point closely aligns with industrial norms at approximately −6 °C. This estimation indicates the practical upper limit attainable through FC for FPH.

### 3.6 Differential scanning calorimetry

DSC analysis was used to examine the thermal properties of FPH at different water content levels. The dried samples were then reconstituted by adding water to achieve the desired dry matter concentrations for DSC analysis. This approach offered detailed observations on temperature fluctuations and enthalpies associated with phase transitions during the heating of FPH within the range of −150 °C to 20 °C. The onset of ice melting, signalling the conclusion of equilibrium freezing, was identified within the temperature span of −22.13 °C to −26.89 °C. Notably, this temperature range closely aligns with the onset temperatures observed in salmon muscles (−29.33 °C) and cod muscles (−33.5 °C) (Tolstorebrov et al., 2014a). As determined by DSC, the initiation of ice melting signifies the conclusion of ice formation under equilibrium conditions, such as during frozen storage. Consequently, reducing the storage temperature of the frozen hydrolysates below this critical value will not lead to subsequent ice formation (Kvangarsnes et al., 2023).

The glass transition temperature varies between −32.39 °C and − 32 °C for different samples with varying water content (Table 6). The glass transition in fish muscles occurs within a broad temperature range from −27 to −90 °C, a phenomenon well-documented in the literature by various studies (Inoue & Ishikawa, 1997; Ohkuma et al., 2008; Shi et al., 2009; Tolstorebrov et al., 2014a). The study by Netto et al. (1998) indicated a glass transition range of −30 to 41 °C for low-moisture FPH, with moisture content ranging from 14 to 0.021 % w.b.

**Table 6:** Thermal properties of FPH concerning different water content.

**Sample name**	**Water content (%)**	**Glass transition temperature (°C)**	**Melting ice (kJ/kg)**	**Freezing point (°C)**	**T**_**m**_	**Unfreezable water %**	**Ice (%)**
Cod hydrolysates	49.3 ± 0.3	−31.5 ± 0.5	92.55 ± 0.6	−9.61 ± 0.1	−26.4 ± 0.4	22.15 ± 0.5	27.4 ± 0.2
	52.6 ± 0.6	−32.5 ± 0.5	105.2 ± 0.5	−8.3 ± 0.2	−26.3 ± 0.5	20.4 ± 0.8	31.5 ± 0.2
	60.5 ± 0.5	−32.73 ± 0.3	144.4 ± 0.6	−5.12 ± 0.12	−26.2 ± 0.4	17.2 ± 0.9	43.2 ± 0.2
	67.5 ± 0.5	−31.6 ± 0.6	176.2 ± 1.8	−3.24 ± 0.14	−21.9 ± 0.9	15.3 ± 0.7	52.8 ± 0.5
	75.42 ± 0.42	−32.7 ± 0.3	207 ± 2	−1.89 ± 0.12	−24.7 ± 0.5	12.4 ± 0.5	61.9 ± 0.6
	87.3 ± 0.3	N/D	284.25 ± 1.25	−1.4 ± 0.1	−21.5 ± 0.6	2.25 ± 0.3	85.1 ± 0.4
	91.5 ± 0.5	N/D	311.5 ± 1.5	−0.545 ± 0.1	−23.7 ± 0.8	0.95 ± 0.2	93.3 ± 0.5

The dry matter significantly influences the freezing point of FPH and tends to decrease with lower moisture content. Utilizing the unfreezable water determination method outlined by Tolstorebrov et al. (2014b), unfreezable water was identified in every sample where ice formation was observed. The ratio of unfrozen water was calculated to fall within the range of 1.11 % to 22.6 %. Nevertheless, in the current study, the glass transition was not observed in FPH samples with a water content exceeding 80 % w.b.

**Fig. 4:**
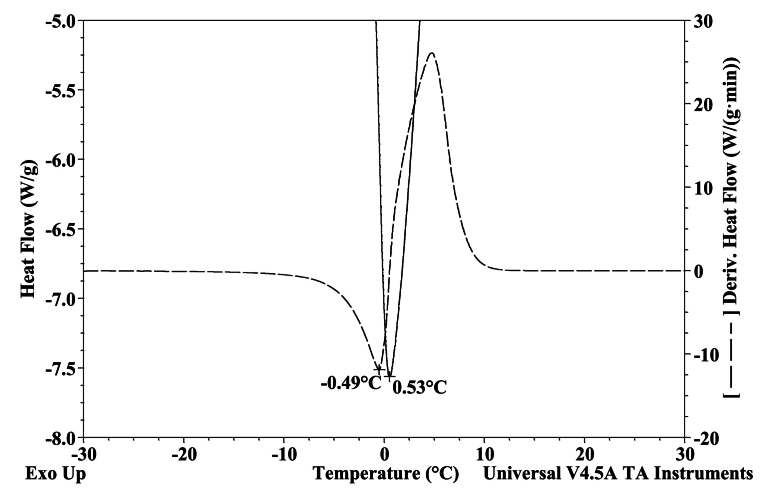
DSC curve for cod hydrolysates with water content of 91.9 %.

The dry matter significantly influences the freezing point of FPH and tends to decrease with lower moisture content. The freezing point of FPH samples with elevated water content was determined by analysing the time derivative of the heat flow, as depicted in Fig. 4. A similar behaviour was noted, mirroring the characteristics observed in pure water.

**Fig. 5:**
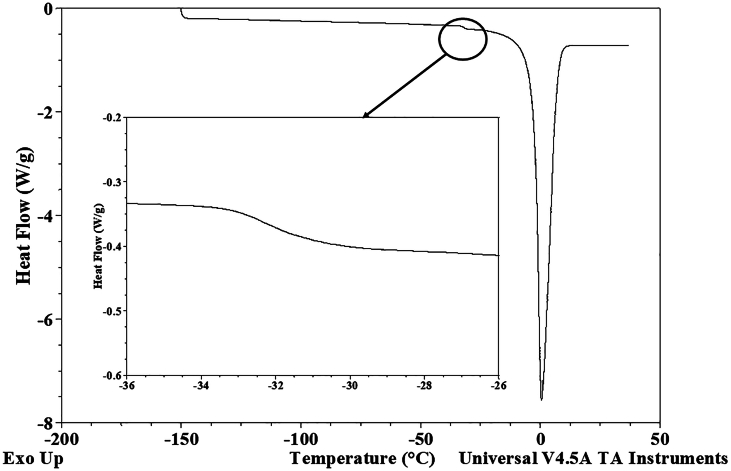
Determination of Phase transitions in FPH.

A melting shoulder was observed near the onset of ice melting, as illustrated in Fig. 5. The distinctive shape of the shoulder, marked by a step change in heat flow, closely resembles the characteristics of a glass transition. A second melting peak is typical for saturated solutions of salts near the eutectic point. Free amino acids can create eutectic solutions with salts at specific temperatures below the solution's freezing point. Furthermore, the eutectic melting peak has been highlighted as an indicator of the limited cryo-protective effect exhibited by specific amino acids (Auwal et al., 2017).

The FPH exhibits moderate hydrolysis Table 2, with a substantial portion of its proteins existing as free amino acids, peptides, and polypeptides. In such instances, all components within the FPH solution exist in a crystalline-solid state below the “end of freezing point,” and the maximal FC is not attained (Kvangarsnes et al., 2023).

### 3.7 High temperature heat pump

**Fig. 6:**
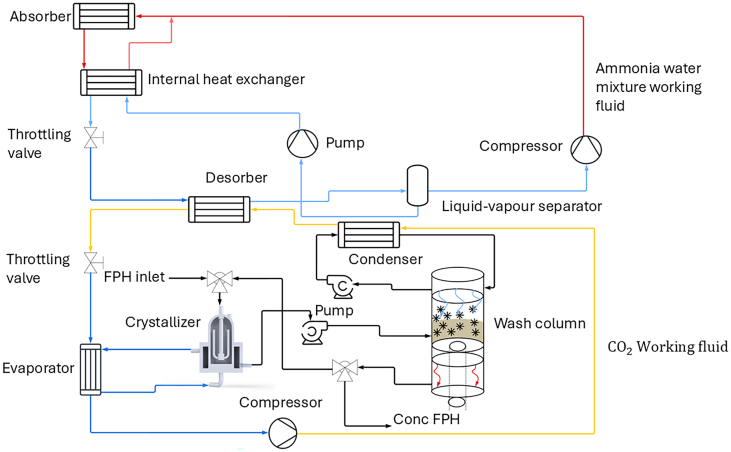
Schematic diagram of a temperature heat pump.

The process is depicted in Fig. 6 to address the integration of a high-temperature heat pump. The FC system has two main components: a crystallizer and a wash column. The crystallizer facilitates ice formation, while the wash column removes the ice from the concentrated solution.

The heat extracted from the crystallizer at −10 °C is efficiently utilized by a condenser to melt the ice at 0 °C. The water obtained from ice melting can then be employed in hydrolysis. The CO_2_ gas exiting the condenser also acts as a heat source for the ammonia-water heat exchanger. This thermal input causes ammonia to transition into a vapor state, separating the vapor from the liquid in the separator. The vaporized ammonia subsequently transfers heat to an absorber operating at two distinct temperature levels: 50 °C and 90 °C.

The high-temperature heat pump is strategically integrated between points 5, 1, and 2, as illustrated in Fig. 1. This placement optimizes energy recovery and enhances overall process efficiency, showcasing the significant potential of heat pump technology in reducing energy consumption for the hydrolysis and FC processes.

## 4 Conclusion

FPH, derived from cod hydrolysates, stands out for its favourable physicochemical and rheological properties, competing with established food proteins. The hydrolysis of cod was carried out using commercial enzymes papain and bromelain, leading to the production of FPH characterized by high protein content and low levels of lipids and salt. FPH contains all essential amino acids, with lysine being a prominent component (FPH before filtration: 49.86 ± 1.58, FPH after polishing: 46.32 ± 3.92, and Before FC: 42.39 ± 15.58).

DSC analysis reveals a maximum glass transition temperature at −32.45 °C, while the freezing point decreases to −9.72 °C. Rheological analysis demonstrates that FPH behaves as a Newtonian fluid up to 44 % w.b., transitioning into a non-Newtonian fluid characteristic of Bingham fluids above this concentration. The study further suggests that a maximum concentration of 43 % can be achieved through FC, potentially reducing energy demand by up to 30 % in the drying process.

Considering the chemical properties of cod hydrolysates throughout the drying process provides valuable insights for designing heat pumps that can achieve the necessary hydrolysis temperature. This approach offers a viable alternative to traditional steam methods, as water, being the primary constituent during hydrolysis, possesses excellent thermal properties. Utilizing heat pumps in place of conventional systems not only ensures efficient temperature control and presents an economically feasible solution, optimizing energy consumption and reducing operational costs. The physicochemical and rheological attributes position FPH as an excellent candidate for concentration through FC, holding immense potential for widespread application in various sectors within the food industry.

## Funding and Acknowledgement

This research was supported by contract no. 245/2021, project acronym 710 SUMAFOOD (sustainable preservation of marine biomasses for an enhanced 711 food value chain). The authors acknowledge the financial support for this project provided by 712 transnational funding bodies, partners of the ERANET BLUE-BIO. Mobility of the research team was supported by 10.13039/501100004787the Research Council of Norway INTPART project Interdisciplinary Education and Research Platform in Cold-Chain of Fish: From Norway to Japan (Pr. Nr: 309841).

## CRediT authorship contribution statement

**Muhammad Umar Khan:** Writing – original draft, Methodology, Investigation, Funding acquisition, Formal analysis, Data curation. **Khalid Hamid:** Writing – review & editing, Conceptualization. **Ignat Tolstorebrov:** Writing – review & editing. **Turid Rustad:** Writing – review & editing, Supervision. **Trygve M. Eikevik:** Writing – review & editing, Supervision. **Manabu Watanabe:** Writing – review & editing, Visualization, Supervision.

## Declaration of competing interest

The authors declare that they have no known competing financial interests or personal relationships that could have appeared to influence the work reported in this paper.

## Data Availability

Data will be made available on request.
